# Marine-derived alginate oligosaccharides as functional modulators for sodium reduction in coated frozen foods: molecular mechanisms, batter matrix engineering, and sensory compensation: a systematic review

**DOI:** 10.3389/fnut.2026.1865128

**Published:** 2026-06-26

**Authors:** Eric Biney, Wanzi Yao, Wenjie Chen, Jiajing Li, Min Wang, Saiyi Zhong, Kit-Leong Cheong

**Affiliations:** 1 College of Food Science and Technology, Guangdong Ocean University, Guangdong Provincial Key Laboratory of Aquatic Product Processing and Safety, Guangdong Province Engineering Laboratory for Marine Biological Products, Guangdong Provincial Engineering Technology Research Centre of Seafood, Guangdong Provincial Engineering Technology Research Center of Prefabricated Seafood Processing and Quality Control, Zhanjiang, China; 2 School of Advanced Agricultural Sciences, Peking University, Beijing, China; 3 College of Coastal Agriculture Sciences, Guangdong Ocean University, Zhanjiang, China

**Keywords:** alginate oligosaccharides, batter systems, food microstructure, marine polysaccharides, salt reduction strategies

## Abstract

Excessive sodium consumption is closely linked to hypertension and cardiovascular illnesses, leading to worldwide initiatives aimed at decreasing salt content in processed foods. Most of the included studies focused on conventional sodium alginate-based hydrocolloid systems, whereas comparatively limited evidence was available for alginate oligosaccharides (AOS) in sodium-reduced, coated, frozen-food applications. Coated frozen foods, including battered fish, chicken nuggets, and fried potato items, significantly contribute to sodium consumption due to the prevalent use of salt for flavor enhancement, batter efficacy, and moisture regulation. Alginate oligosaccharides (AOS), derived from brown seaweed alginate and obtained by depolymerization, have recently attracted interest as multifunctional food hydrocolloids that can influence microstructure, water retention, and flavor perception in reduced-sodium formulations. This systematic analysis assesses peer-reviewed literature published from 2005 to 2025 on the function of alginate or alginate-derived hydrocolloids in sodium-reduction techniques in coated and fried food systems.

## 1 Introduction

### 1.1 Global need for sodium reduction in processed foods

Excessive salt intake constitutes a significant global public health issue. The World Health Organization advises that daily sodium consumption should be restricted to under 2 g; yet, the average intake in numerous nations surpasses this guideline, primarily owing to processed foods (1). Coated frozen foods, such as battered fish, chicken nuggets, shrimp, and fried potato products, significantly contribute to sodium consumption due to salt’s several technological roles, including flavor enhancement, protein solubilization, water binding, and crust development (2). Decreasing sodium in these items is notably difficult as salt affects various facets of product quality concurrently, including as flavor perception, batter viscosity, moisture retention, and crispness (3). The elimination or substitution of salt frequently leads to a lackluster flavor, diminished batter adherence, and subpar texture. As a result, contemporary sodium-reduction efforts increasingly depend on multifunctional compounds that offset these technological deficiencies (3, 4).

### 1.2 Hydrocolloids in batter and frying systems

Hydrocolloids are important in enhancing the quality of fried and coated foods. These polymers modulate viscosity, batter adherence, adhesion, moisture retention, and crust development during frying. Hydrocolloids diminish oil absorption by creating thin surface coatings that serve as diffusion barriers between the frying oil and the food matrix (5). In batter systems, hydrocolloids augment viscosity and viscoelasticity, hence improving coating stability during frying and freezing. They also alter water migration and pore formation during thermal processing, significantly influencing oil absorption and texture (6). Studies indicate that alginate-based coatings can markedly diminish oil absorption and maintain crispness by creating dense hydrophilic films that restrict pore development and moisture evaporation during frying (7). The functional features indicate that hydrocolloids may mitigate the technical and sensory difficulties linked to sodium reduction (8).

### 1.3 Marine-derived alginate oligosaccharides

Alginate is a naturally occurring polysaccharide predominantly derived from brown seaweeds, including *Laminaria*, *Macrocystis*, and *Ascophyllum* (9). Alginate is composed of *β*-D-mannuronic acid (M) and *α*-L-guluronic acid (G) residues organized into homopolymeric and heteropolymeric blocks. Alginate can be transformed into alginate oligosaccharides (AOS) with reduced molecular weight and enhanced solubility via enzymatic or chemical depolymerization (10, 11). Although sodium alginate and alginate oligosaccharides (AOS) originate from the same marine polysaccharide source, they differ substantially in molecular structure and functional properties (12). Sodium alginate is a high-molecular-weight linear copolymer composed of *β*-D-mannuronic acid (M) and *α*-L-guluronic acid (G) residues, widely recognized for its thickening, gelling, emulsifying, and water-binding capabilities in food systems (13). Due to their shorter chain lengths, higher solubility, and greater molecular mobility, AOS exhibit distinct biological and physicochemical functionalities, including enhanced antioxidant, prebiotic, and flavor-modulating properties (14). While conventional sodium alginate has been extensively investigated for improving batter rheology, moisture retention, and oil reduction in fried foods, direct evidence regarding the application of AOS in sodium-reduced coated frozen food systems remains comparatively limited. Therefore, it is important to distinguish findings derived from conventional alginate-based hydrocolloids from those specifically attributed to AOS when evaluating their potential roles in sodium-reduction strategies (15). These attributes render them advantageous for the formulation of edible coatings, films, and complex food matrices (16, 17).

### 1.4 Alginate-based matrix engineering in fried foods

**Figure 1:**
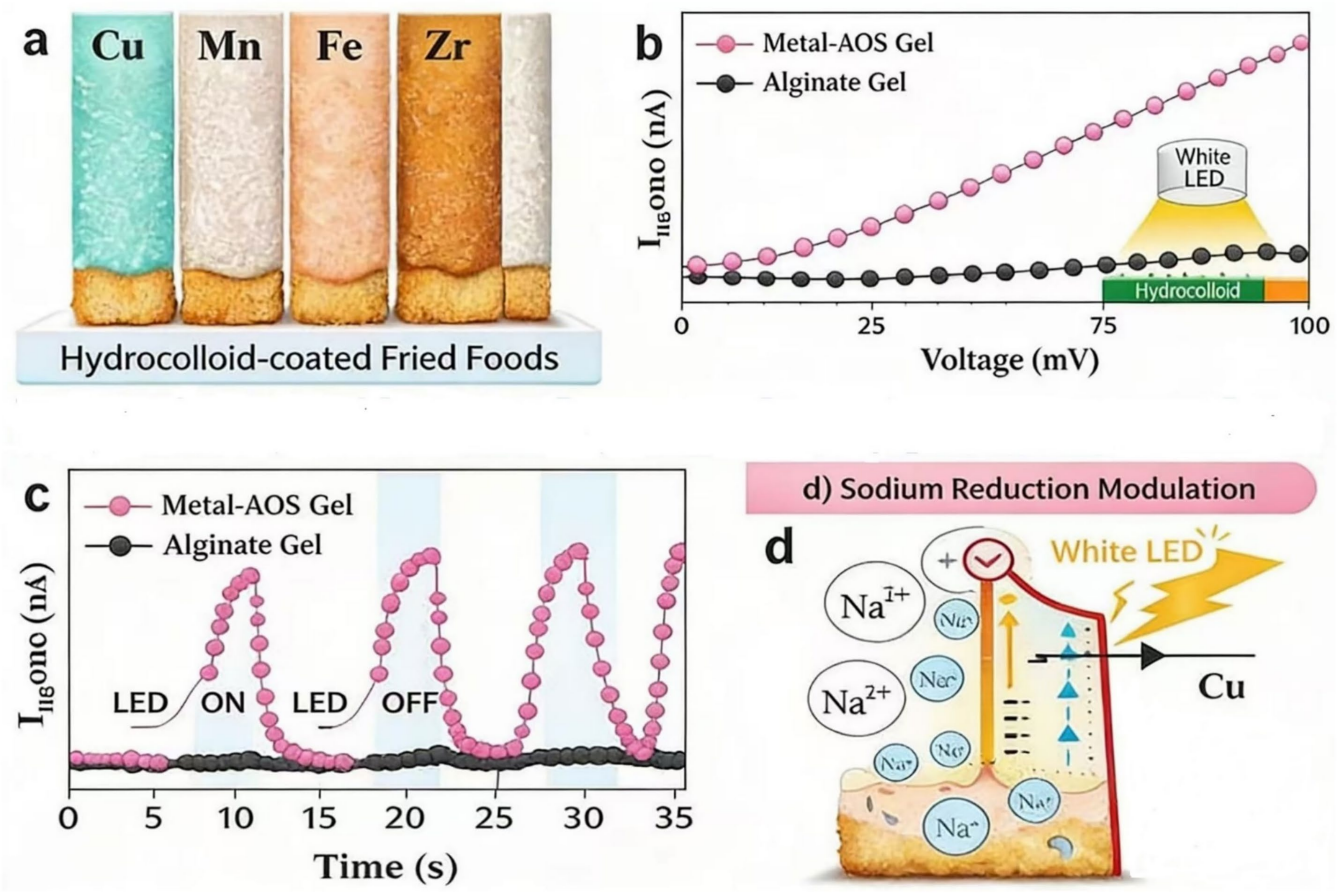
**(a)** Optical image of the prepared MAlgGel samples placed on top of a printed logo. **(b)** Photoresponse of MnAlgGel and AlgGel upon exposure to white LED. **(c)** Time-resolved photoresponse of the MnAlgGel and AlgGel photodetector with a channel length of 1 cm. **(d)** Band diagram of the MnAlgGel/Cu electrode interface. Second row: Injectable Interleukin 2 (IL2) loaded into Ni2 + −alginate microspheres for cancer therapy (18).

Alginate’s capacity to create gel networks in the presence of divalent cations constitutes the predominant category of ions that interact with alginate to establish cross-linked structures. The subsequent M^2+^ ions are purportedly capable of crosslinking alginate: Mg^2+^, Ca^2+^, Sr^2+^, Ba^2+^, Mn^2+^, Co^2+^, Ni^2+^, Cu^2+^, Zn^2+^, Cd^2+^, Pb^2+^, UO^22+^, as shown in Figure 1 (18). These networks improve moisture retention and reduce oil penetration during frying (19). Studies conducted by Ying Li et al. (15) demonstrate that alginate coatings significantly reduce oil uptake in fried potato products by modifying the crust’s microstructure and reducing water evaporation. Alginate-based coatings not only decrease oil consumption but also inhibit the production of detrimental chemicals, such as acrylamide, during frying, all while preserving sensory attributes (15). Recent research also indicates that alginate in starch-based batter systems enhances viscosity, improves batter adhesion, and enhances crispness and overall appeal in fried foods (20, 21). These functional properties indicate that alginate-derived polymers may serve as multifunctional tools in sodium reduction strategies (22).

### 1.5 Rationale for the review

Although extensive study has been conducted on hydrocolloids and edible coatings in fried foods, the specific potential of marine-derived alginate oligosaccharides for sodium reduction in coated frozen meals has not been systematically synthesized (22). Comprehending the molecular and microstructural mechanisms by which AOS affect batter systems and sensory perception may enable the development of healthier, reduced-sodium frozen foods. This systematic review seeks to consolidate existing evidence regarding the utilization of alginate and alginate oligosaccharides in coated and fried foods, concentrating on three principal aspects: (I) Molecular and physicochemical mechanisms of alginate-based hydrocolloids, (II) Batter matrix engineering and microstructural modulation, (III) Sensory compensation strategies facilitating sodium reduction (23, 24).

### 1.6 Conceptual framework of alginate-based sodium reduction in coated frozen foods

**Figure 2:**
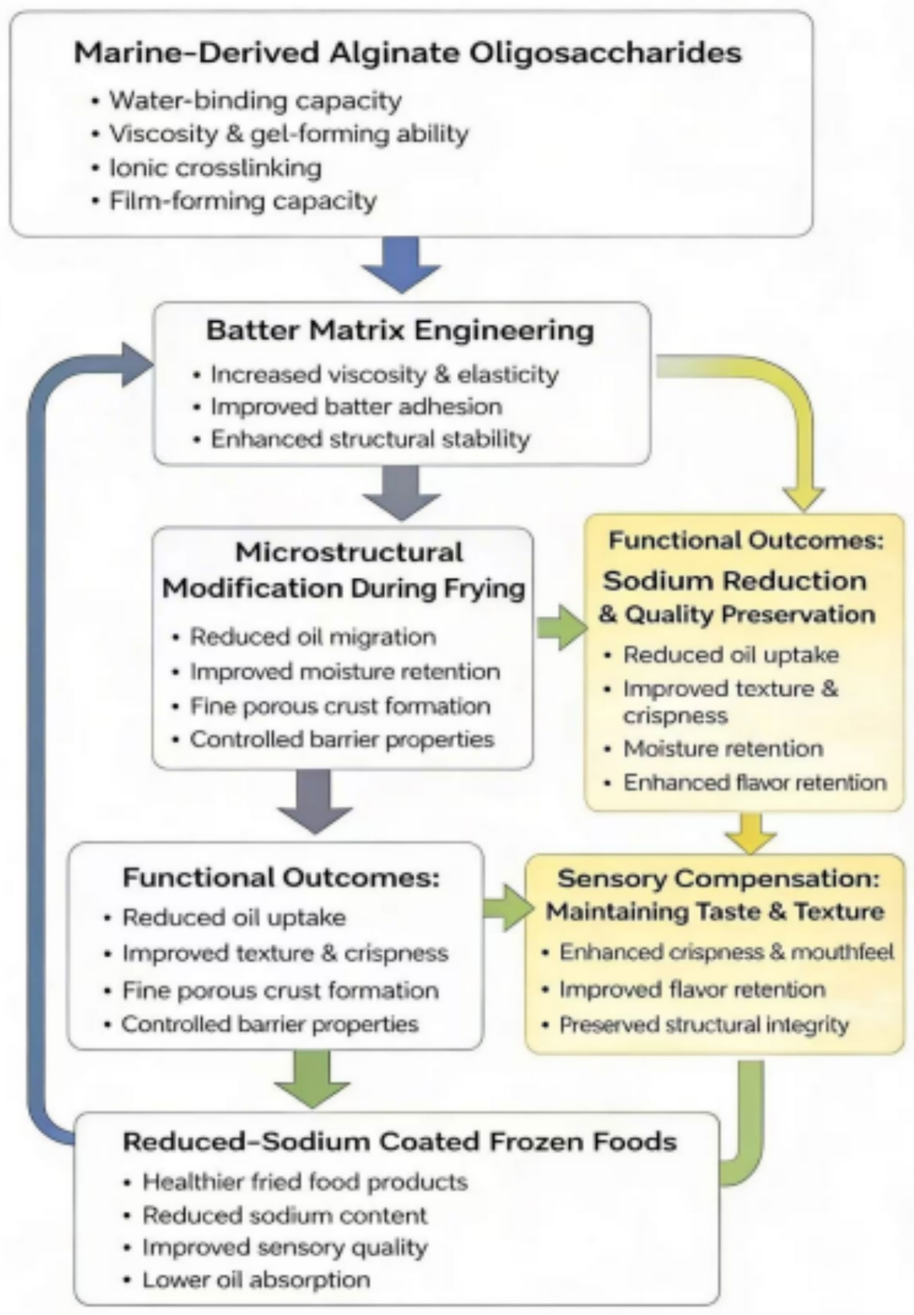
Conceptual framework for the function of marine-derived alginate oligosaccharides in salt reduction in coated frozen meals.

We propose the conceptual framework depicted in Figure 2, which delineates the interrelationships among functional ingredients, processing methods, and quality outcomes in reduced-sodium-coated frozen food systems. The framework elucidates the mechanisms by which marine-derived alginate oligosaccharides (AOS) act as modulators, affecting batter matrix characteristics, microstructural changes during frying, and ultimately the qualitative attributes of the final product (25). The framework illustrates how integrating AOS as a principal functional driver alters battery system processes, including viscosity development, gel formation, and coating adhesion. These physicochemical alterations subsequently affect microstructural processes during frying, including moisture retention, pore development, and oil migration (25, 26). Consequently, these mechanisms influence essential functional outcomes, such as less oil absorption, greater texture and crispness, and improved flavor retention, all of which provide successful sodium reduction without sacrificing sensory quality. The paradigm additionally includes formulation and processing variables, such as hydrocolloid concentration, batter composition, and frying conditions, as components of the overarching environment that influences system performance (27). These parameters affect the efficacy with which AOS can alter structural and sensory characteristics of coated food products. While not explicitly modeled, external factors such as consumer preferences, regulatory sodium-reduction targets, and advancements in food processing technologies are recognized as significant contextual variables that may impact future developments in this domain. Although the framework is depicted as a linear progression for clarity, it is crucial to acknowledge that food systems are fundamentally dynamic and interrelated (28). The correlations among batter composition, microstructural development, and sensory experience are frequently nonlinear and may entail feedback mechanisms (29). Changes in moisture retention can concurrently affect oil absorption, texture, and flavor release, thereby influencing customer perception and product reformulation strategies (30). This systematic review considers AOS incorporation as the principal functional driver, analyzing its effects throughout various stages of batter matrix engineering, frying-induced microstructural alterations, and final product quality outcomes. This conceptual framework provides a cohesive understanding of the strategic application of marine-derived alginate oligosaccharides in the development of reduced-sodium-coated frozen foods, thereby enhancing both technological and sensory attributes (31).

## 2 Materials and methods

### 2.1 Literature search and study selection

A systematic literature analysis was performed to find pertinent research investigating the role of marine-derived alginate and its oligosaccharides (AOS) in salt reduction techniques for coated and fried food systems. The search method was formulated in accordance with the PRISMA framework for systematic reviews, as outlined in the PRISMA 2020 Statement (32). The search strings were generated utilizing combinations of three fundamental thematic concepts: alginate or alginate oligosaccharides, sodium reduction or salt substitution, and coated or fried food systems. To guarantee thorough retrieval of pertinent material, many synonyms and associated terms were incorporated, including hydrocolloids, batter systems, edible coatings, oil absorption, texture alteration, and sensory acceptance (33). All phases of the systematic review procedure, encompassing research identification, screening, eligibility evaluation, data extraction, and qualitative synthesis, were conducted in alignment with the PRISMA methodological framework. A comprehensive PRISMA checklist is included in the supplementary materials accompanying this evaluation. Prior to the preliminary search, it was acknowledged that pertinent scientific literature is disseminated throughout many international bibliographic databases commonly utilized in food science and nutrition research. Consequently, many electronic resources were accessed to guarantee thorough coverage of the existing literature. The sources comprised Web of Science (WoS), Scopus, PubMed, ScienceDirect, and Google Scholar. Web of Science, indexing over 34,000 peer-reviewed journals across many scientific disciplines, was chosen as the major database because of its comprehensive citation indexing, superior journal coverage, and dependability as a source of peer-reviewed literature (34).

**Figure 3:**
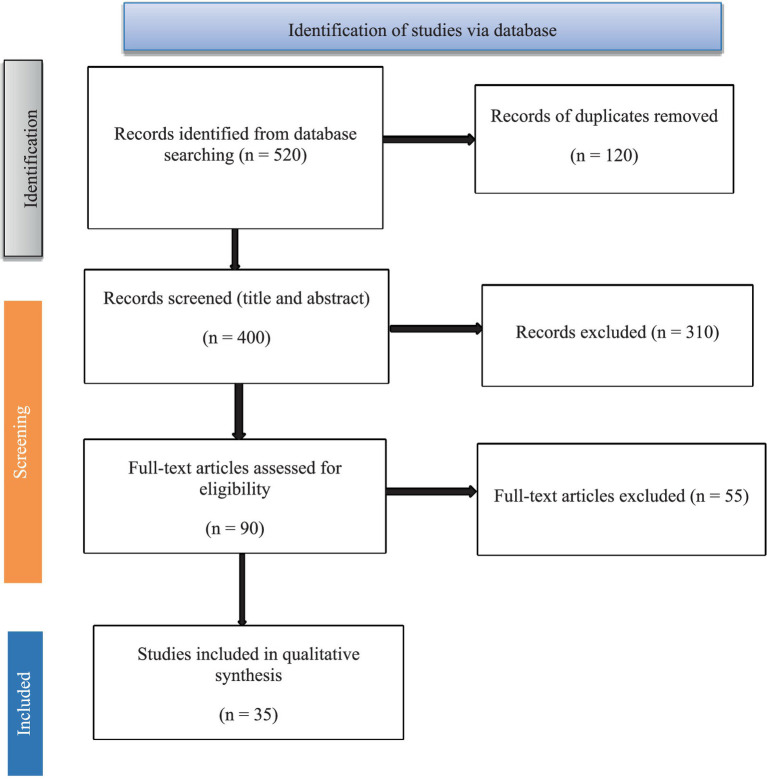
Present’s the study identification, screening, and selection process followed in this systematic review according to the PRISMA 2020 Guidelines. The diagram illustrates the systematic filtering procedure used to identify relevant studies investigating the role of marine-derived alginate oligosaccharides and related hydrocolloids in sodium reduction strategies for coated frozen foods.

Supplementary databases were utilized to enhance the search and guarantee the inclusion of possibly pertinent studies not included in WoS. The literature search was conducted between November and December 2025, and studies published up to December 2025 were considered for inclusion. Search criteria were generated based on the review’s principal themes: alginate-derived hydrocolloids, sodium-reduction techniques, and batter or coating systems utilized in fried or frozen foods. Boolean operators were employed to amalgamate search keywords and optimize the retrieval of pertinent articles. The search strategy encompassed the subsequent keywords and combinations: (“alginate” OR “alginate oligosaccharide” OR “sodium alginate” OR “marine polysaccharide”) AND (“sodium reduction” OR “salt reduction” OR “low sodium”) AND (“batter” OR “coating” OR “breadcrumb” OR “edible coating” OR “hydrocolloid”) AND (“fried food*” OR “coated food*” OR “frozen food*” OR “fried product*”). The entire procedure of study identification, screening, eligibility assessment, and inclusion is depicted in the PRISMA flow diagram (Figure 3), with a comprehensive overview of inclusion and exclusion criteria provided in Table 1. Initially, titles and abstracts were manually evaluated to determine their pertinence to the review’s aims. Relevant studies were later assessed in full text to verify eligibility. Upon using these criteria, qualifying studies were incorporated into the final synthesis. Abstracts that met these requirements were imported into EndNote 21 and evaluated through a multi-stage procedure derived from the seven-step methodology (see Table 2), and duplicate entries were removed. The remaining studies underwent a two-phase screening procedure.

**Table 1:** Inclusion and exclusion criteria for the selection of articles.

Criteria category	Inclusion criteria	Exclusion criteria
Publication type	Peer-reviewed journal articles	Conference abstracts, editorials, book chapters, theses, reports without full experimental data
Publication period	Studies published between 2005 and 2025	Studies published before 2005
Language	Articles published in English	Non-English publications
Research focus	Studies investigating alginate, alginate oligosaccharides (AOS), or related hydrocolloids in food systems	Studies focusing on non-food applications, such as environmental, agricultural, or industrial uses
Application area	Research examining batter systems, coatings, breadcrumb matrices, or edible films in fried or coated foods	Studies focusing on pharmaceutical, biomedical, or drug delivery applications of alginate
Food system studied	Studies involving fried foods, coated foods, or frozen coated food products (e.g., fried fish, chicken nuggets, battered vegetables)	Studies unrelated to fried or coated food systems
Functional outcomes	Studies reporting sodium/salt reduction strategies, rheological properties, microstructural characteristics, oil uptake, or sensory attributes	Studies that do not evaluate functional, structural, or sensory properties relevant to sodium reduction
Study design	Experimental or analytical studies providing measurable data on hydrocolloid functionality in food matrices	Studies lacking experimental data or quantitative analysis
Relevance to review topic	Studies directly related to alginate-based hydrocolloids for improving batter properties, microstructure, or sensory quality under reduced sodium conditions.	Studies unrelated to hydrocolloids, batter engineering, or sodium reduction strategies

**Table 2:** Seven-step EndNote de-duplication sequence.

Step	De-duplication procedure	Description	Purpose
1	Automatic duplicate detection	The Find duplicates function in EndNote was used to identify records with identical author names, publication year, and titles.	Rapid identification of obvious duplicate records across databases.
2	Title-based comparison	References were sorted alphabetically by article title, and records with identical or highly similar titles were manually inspected.	To detect duplicates not captured by automated matching due to minor metadata differences.
3	DOI verification	Entries were sorted by Digital Object Identifier (DOI), and records with identical DOIs were identified and consolidated.	DOI matching provides highly accurate duplicate detection.
4	Author and year cross-check	Records were organized by first author and publication year, and suspected duplicates were manually verified.	To detect duplicates where titles differ slightly due to formatting differences
5	Journal and volume comparison	References were sorted by journal name, volume, and issue number.	To identify duplicates arising from database indexing inconsistencies
6	Abstract and keyword comparison	Records with highly similar abstracts or keywords were manually reviewed.	To capture duplicates that differ in titles but represent the same study
7	Final manual inspection	The entire reference library was manually reviewed to identify any remaining duplicates or incomplete records.	Ensures a fully cleaned dataset before screening stages.

#### 2.1.1 PICOS framework

To improve methodological transparency and ensure alignment with the Preferred Reporting Items for Systematic Reviews and Meta-Analyses (PRISMA 2020) guidelines, the review question, search strategy, and eligibility criteria were structured using the PICOS (Population, Intervention, Comparator, Outcomes, and Study Design) framework (35). Population (P): Coated and fried food systems, including fish products, poultry products, potato-based products, seafood products, and plant-based fried foods. Intervention (I): Application of alginate, alginate oligosaccharides (AOS), or alginate-based hydrocolloid formulations as functional ingredients in sodium-reduced batter, coating, or fried food systems. Comparator (C): Conventional batter and coating formulations, standard sodium-containing products, or control treatments without alginate incorporation. Outcomes (O): Technological, physicochemical, and sensory outcomes related to sodium reduction, including batter rheology, viscosity, coating adhesion, moisture retention, oil absorption, texture profile, crust microstructure, sodium reduction efficiency, flavor perception, consumer acceptability, and overall product quality. Study Design (S): Peer-reviewed experimental studies, food formulation studies, sensory evaluation studies, and mechanistic investigations examining the functional effects of alginate or alginate-derived hydrocolloids in coated and fried food systems. The PICOS framework guided the selection of studies included in this review and ensured a systematic evaluation of the available evidence regarding the application of alginate-based hydrocolloids for sodium reduction in coated frozen food products.

### 2.2 Data extraction, analysis, preparation, and synthesis procedure

Subsequent to the comprehensive eligibility evaluation, the studies that satisfied the established inclusion criteria were methodically analyzed to obtain pertinent material for qualitative synthesis. The investigations were classified based on essential criteria and outcome domains, such as hydrocolloid type, food system examined, sodium-reduction approach, batter formulation parameters, rheological properties, microstructural features, oil absorption, and sensory qualities (36). This classification facilitated a systematic evaluation of the functional roles of alginate-based hydrocolloids in batter systems utilized for coated and fried foods. Data extraction was performed utilizing a predetermined, consistent template to guarantee uniformity and transparency during the review process. The extracted information encompassed publication details (authors, year of publication, and journal), type of hydrocolloid utilized (e.g., sodium alginate or alginate oligosaccharides), concentration or formulation employed in the batter system, food product examined (such as battered fish, chicken products, or fried vegetables), experimental conditions, and documented outcome measures. The principal outcome factors encompassed batter rheology (such as viscosity and viscoelastic characteristics), oil absorption during frying, moisture retention, crust microstructure, texture profile analysis, and sensory assessment results pertinent to sodium-reduced formulations.

Numerical values were extracted from graphical representations of experimental data using WebPlotDigitizer (version 5.2) to enable quantitative analysis of the reported findings. When applicable, outcome measures, including oil uptake, moisture retention, or textural parameters, were transformed into percentage changes compared to control samples, facilitating standardized comparisons across studies with varying experimental settings (37). The information obtained from each qualifying study was systematically structured and summarized in a tabular style, facilitating a comparative analysis of hydrocolloid formulations, food matrices, processing parameters, and documented functional outcomes. This method enabled the recognition of patterns in the utilization of alginate-based hydrocolloids for batter matrix formulation, oil migration regulation during frying, and sensory compensatory strategies in reduced-sodium coated meals. Owing to the considerable heterogeneity among the studies analyzed, characterized by variations in dietary systems, hydrocolloid formulations, sodium-reduction techniques, experimental methodologies, and reported outcome variables, a quantitative meta-analysis was deemed unsuitable (38). The findings were synthesized through a qualitative narrative technique, highlighting descriptive comparison and theme analysis across studies. The narrative synthesis concentrated on identifying prevalent functional mechanisms, novel technical strategies, and research deficiencies with the utilization of marine-derived alginate oligosaccharides and associated hydrocolloids in sodium-reduced batter systems. Special emphasis was placed on the function of these biopolymers in altering batter rheology, microstructural development during frying, oil absorption characteristics, and sensory evaluation in coated frozen meals. The qualitative synthesis offers a cohesive assessment of the existing body of information. It underscores prospective avenues for further investigation into alginate oligosaccharides as functional modulators in sodium-reduced coated food systems.

### 2.3 Risk of bias assessment

Risk of bias for the included studies was assessed using a modified version of the Revised Cochrane Risk of Bias tool for Randomized Trials (RoB 2) (39). Adapted for experimental food science and sensory evaluation studies. The assessment considered five domains: (i) experimental design and randomization, (ii) deviations from intended interventions, (iii) missing outcome data, (iv) outcome measurement, and (v) selection of reported results. Each domain was classified as low risk, some concerns, or high risk of bias. Two reviewers independently evaluated all included studies, and disagreements were resolved through discussion or consultation with a third reviewer where necessary. The overall risk-of-bias results were visualized using the RobVis tool (40). Overall, most studies demonstrated moderate methodological quality. Common limitations included insufficient reporting of randomization procedures, limited replication, and incomplete sensory evaluation details. Nevertheless, the included studies provided consistent evidence regarding the functional role of alginate and alginate oligosaccharides (AOS) in sodium reduction, batter matrix modification, and sensory optimization in coated frozen foods (41).

### 2.4 Study protocol

The methodology for this systematic review was developed in accordance with the methodological framework for systematic reviews proposed in the Cochrane Handbook for Systematic Reviews and reported following the PRISMA 2020 guidelines (32) to ensure transparency and reproducibility. The review protocol was adapted for food science and nutrition research focusing on marine-derived alginate and alginate oligosaccharides (AOS) in sodium-reduced coated frozen foods. The study selection, screening, data extraction, and qualitative synthesis procedures were predefined prior to the literature search to minimize bias and improve methodological consistency.

### 2.5 Data synthesis

Data extracted from the included studies comprised publication year, food product type, alginate or alginate oligosaccharide (AOS) formulation, hydrocolloid concentration, frying or coating conditions, rheological properties, oil absorption, moisture retention, texture parameters, sensory outcomes, and sodium-reduction effects. Outcome data were summarized as reported values or relative changes compared with control formulations. Methodological limitations and potential sources of bias across studies were also evaluated to identify inconsistencies and research gaps within the current literature. Due to substantial heterogeneity in food matrices, experimental designs, hydrocolloid systems, and analytical methods, a quantitative meta-analysis was not performed. Instead, findings were synthesized qualitatively using structured narrative and comparative approaches. Data management was conducted using Microsoft Excel, and risk-of-bias visualizations were generated using the RobVis application (39).

## 3 Results and discussion

### 3.1 Characteristics of the included studies

The systematic search performed for this review initially obtained 520 documents from designated databases. Following the elimination of 120 duplicate entries, a total of 400 studies underwent title and abstract screening. At this step, 310 publications were removed according to established eligibility criteria, which included irrelevance to alginate-based systems, non-food uses, lack of sodium-reduction context, or absence of evaluation of batter/coating systems. Ninety full-text publications were subsequently evaluated for eligibility. Fifty-five studies were removed after thorough examination due to inadequate reporting of functional outcomes (e.g., rheological, sensory, or oil uptake data), insufficient emphasis on coated or fried food systems, or the lack of explicit hydrocolloid-related interventions. In all, 35 studies fulfilled all inclusion criteria and were selected for qualitative synthesis. The investigations were classified into specific research types according to the established methodological approaches (42). Approximately 31% of the research were empirical-quantitative, concentrating on quantifiable physicochemical and functional factors including viscosity, oil absorption, and texture. Approximately 29% were empirical-qualitative, focusing on descriptive evaluations, especially in sensory analysis and product characterisation. 23% of research utilized mixed-method techniques, combining instrumental analysis with sensory evaluation, whilst 17 % were conceptual or review-based, examining the mechanisms and applications of alginate-based hydrocolloids in food systems.

The historical distribution of the papers reveals that most were published post-2015, with a significant rise noted from 2017, indicating an increasing scientific and industry focus on sodium reduction and healthier food reformulation tactics. This trend corresponds with heightened global focus on decreasing dietary salt consumption and enhancing the nutritional quality of processed foods (43). The studies were primarily sourced from Asia and Europe, with supplementary contributions from North America and minimal representation from other locations (15). A multitude of studies concentrated on single-country experimental contexts, whilst a lesser number examined comparative or multi-regional evaluations of food systems and processing methodologies. The selected literature predominantly consisted of original research publications, with a lesser number of review papers and methodological studies (44). The experimental research primarily concentrated on particular food systems, such as battered fish, poultry items, and fried plant-based foods, which are typically linked to elevated sodium and oil levels (45). While all research examined hydrocolloid functioning in food systems, only a small fraction particularly focused on alginate oligosaccharides (AOS) (46). Although the broader alginate literature demonstrates strong technological potential, direct evidence regarding AOS-specific applications in sodium-reduced coated frozen foods remains limited and warrants further investigation. Most studies focused on conventional sodium alginate or composite hydrocolloid systems, indicating a research gap in the targeted application of AOS for sodium reduction in coated frozen foods (47). Moreover, despite the examination of numerous food quality indicators, such as batter rheology, oil absorption, texture, and sensory characteristics, less than 50 % of the research included thorough sensory evaluation or consumer-oriented analysis (48). This indicates that although technological feasibility has been thoroughly examined, consumer perceptions and acceptance of reduced-sodium formulations are still insufficiently researched.

The distribution and characteristics of the included papers underscore a burgeoning and dynamic research domain, with a heightened focus on the integration of functional ingredient design, food matrix engineering, and health-oriented reformulation methodologies (49). The small number of studies particularly addressing AOS highlights the necessity for additional targeted study to fully harness their potential in sodium-reduced coated food systems (50).

### 3.2 Risk of Bias assessment in human studies

Risk-of-bias assessment was conducted for all included studies using the modified RoB 2 framework (39). Overall, most studies demonstrated moderate-to-high risk of bias across several methodological domains (Table 3). The highest concerns were associated with deviations from intended interventions, incomplete outcome data, and selective reporting of results. Several studies lacked detailed descriptions of randomization procedures, replication strategies, and standardized sensory evaluation protocols, which may have affected the reliability and comparability of reported outcomes. In contrast, the majority of studies showed low risk of bias in outcome measurement, as physicochemical, rheological, and sensory analyses were generally conducted using standardized and validated analytical methods. Studies involving sensory and consumer evaluations frequently exhibited methodological limitations related to panel size, blinding procedures, and reporting transparency. For crossover and repeated-measure experimental designs, additional concerns were identified regarding treatment sequencing and carryover effects, particularly where intervention standardization and washout conditions were insufficiently described. Despite these limitations, the included studies consistently demonstrated the functional potential of alginate and alginate oligosaccharides (AOS) in improving batter structure, reducing oil absorption, enhancing moisture retention, and maintaining sensory acceptability in reduced-sodium coated food systems. Overall, the findings highlight the need for future studies employing more rigorous experimental designs, standardized sensory methodologies, improved statistical reporting, and transparent intervention protocols to strengthen the evidence base for marine-derived hydrocolloids in sodium-reduction applications.

**Table 3:** Risk-of-bias summary for included parallel-group trials.

Risk-of-bias domain	Low risk	Some concerns	High risk	Main observed limitations
Experimental design and randomization	Few studies clearly described proper randomization and replication procedures	Several studies partially described allocation or replication methods	Multiple studies lacked sufficient details regarding randomization and experimental allocation	Inadequate reporting of randomization and limited replication strategies
Deviations from intended interventions	Most studies maintained relatively consistent batter formulations and frying conditions	Minor inconsistencies in intervention implementation were occasionally observed	Few studies inadequately described intervention standardization	Limited reporting of processing consistency and treatment control
Missing outcome data	Most physicochemical datasets were complete	Some studies provided incomplete variability or replicate information	A small number of studies omitted certain outcome measurements	Incomplete reporting of experimental replicates and variability measures
Outcome measurement	Majority of studies used validated analytical methods for rheology, texture, oil uptake, and microstructure	Minor methodological details occasionally missing	Few studies lacked sufficient analytical descriptions	Limited methodological transparency in some analytical procedures
Selection of reported results	Several studies comprehensively reported all measured outcomes	Some studies selectively emphasized favorable findings	Few studies inadequately reported non-significant results	Potential selective reporting of functional or sensory outcomes
Sensory evaluation bias	Limited number of studies employed standardized sensory protocols with trained panels and blinding	Many studies provided incomplete sensory methodological details	Several studies lacked adequate sensory design descriptions	Small panel sizes, absence of blinding, and limited assessor training details
Statistical analysis and replication	Some studies applied appropriate statistical analyses with sufficient replication	Several studies lacked detailed statistical reporting	Few studies used insufficient replication or unclear statistical procedures	Inadequate statistical transparency and limited replication

### 3.3 Temporal distribution

**Figure 4:**
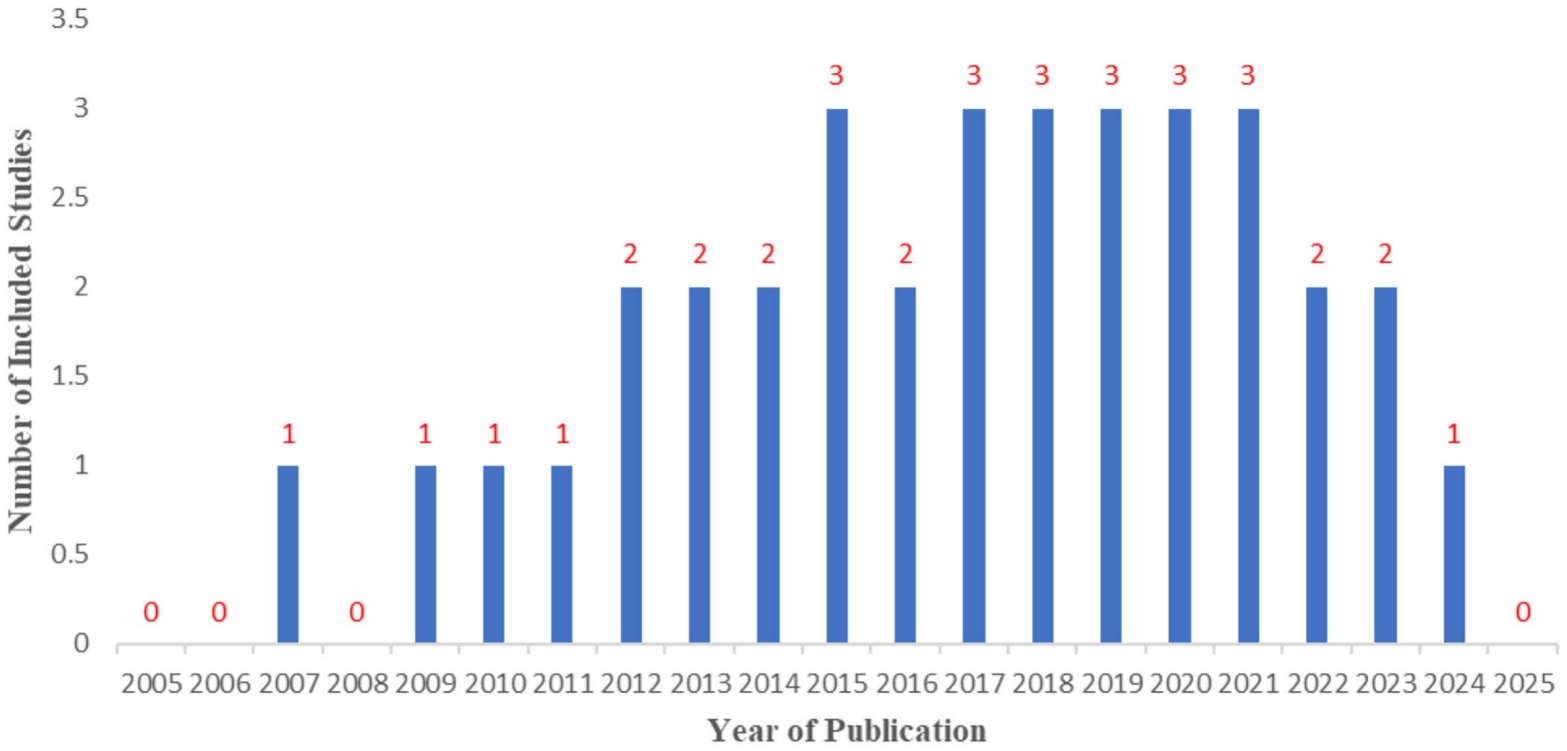
Distribution of included studies by year.

The temporal and geographical distribution of the studies included in this systematic review as presented in Figure 4. A total of 35 studies were analyzed, and their yearly contributions were expressed as percentages to better illustrate research trends over time. The percentage distribution shows that research activity in this field has increased progressively over the last two decades. No studies were identified during 2005–2006, 2008, and 2025 (0%), indicating an absence of research during these years. The earliest contributions appeared in 2007 and 2009, each accounting for 2.9% of the total studies. Between 2010 and 2011, each year contributed 2.9%, followed by a moderate increase in 2012, 2013, 2014, and 2016, each representing 5.7% of the included studies. A notable increase occurred in 2015, accounting for 8.6% of total publications. The most significant growth in research activity occurred during 2017–2021, with each year contributing 8.6%, totaling approximately 42.9% of all included studies. This period represents the peak phase of research, reflecting intensified global interest in sodium reduction and functional hydrocolloid applications in fried and coated food systems. In the most recent period (2022–2023), each year contributed 5.7%, while 2024 accounted for 2.9%, indicating a slight decline but continued research activity. Overall, more than 85% of the included studies were published after 2011, highlighting the fields relatively recent, rapidly growing nature.

### 3.4 Geographical distribution of included studies

The geographical distribution of the included studies reveals a strong regional concentration, with research predominantly conducted in Asia and Europe, and comparatively fewer studies from other regions. Approximately 40–45% of the studies originated from Asia, with major contributions from China, South Korea, and Japan. This dominance is likely due to the availability of marine resources, strong research capacity in food science, and high consumption of fried and coated food products in these countries. Europe accounted for approximately 30–35% of the included studies, with key contributions from Spain, the United Kingdom, Italy, and Denmark. According to Trumbo et al. (51), the region has been largely driven by public health policies aimed at sodium reduction and by advances in food reformulation technologies. North America contributed approximately 15–20% of the studies, primarily from the United States and Canada. Studies from this region focused mainly on industrial applications, food processing innovations, and product development (52). In contrast, limited representation (less than 10%) was observed from regions such as South America and Africa, with only a few studies identified from countries like Brazil and South Africa. This underrepresentation suggests potential gaps in research capacity or in prioritization in these regions, despite the relevance of sodium-reduction strategies (28). The combined temporal and geographical analysis indicate that research on alginate-based hydrocolloids for sodium reduction in coated frozen foods is both rapidly evolving and regionally concentrated. The significant increase in studies after 2015, particularly during 2017–2021, reflects growing global attention toward health-driven food reformulation and functional ingredient innovation (53). However, the included studies represented multiple geographical regions, with the majority originating from Asia and Europe, alongside contributions from North America and selected studies from other regions. This distribution reflects the growing global interest in sodium-reduction strategies and alginate-based food applications.

### 3.5 Molecular mechanisms of alginate in sodium reduction

The findings from the included studies demonstrate that alginate and alginate-derived hydrocolloids function as multifunctional structural modifiers in batter and coating systems, influencing sodium reduction primarily through physicochemical and microstructural mechanisms rather than direct salt replacement (54, 55). Across the analyzed literature, three principal molecular mechanisms were consistently identified: ionic gel formation, water-binding capacity, and surface film formation during frying. Together, these mechanisms significantly alter mass transfer dynamics, crust formation, and flavor perception in coated food products (56). One of the most fundamental mechanisms underlying alginate functionality is its ability to form ionically cross-linked gels in the presence of divalent cations, particularly calcium ions (57). Alginate is composed of *β*-D-mannuronic acid (M) and *α*-L-guluronic acid (G) residues arranged in block-like sequences. The G-block regions interact strongly with calcium ions via the well-established “egg-box” model of ionic cross-linking, leading to the formation of three-dimensional gel networks, as shown in (58). These networks increase structural rigidity within batter matrices and enhance the formation of cohesive coating layers during frying. The resulting gel structures improve mechanical stability and reduce structural collapse during thermal processing (59, 60).

In addition to gel formation, alginate exhibits a high water-binding and hydration capacity, which significantly influences moisture dynamics within fried food systems (61). The hydrophilic carboxyl groups along the alginate polymer chain facilitate strong interactions with water molecules, thereby increasing water retention within the batter matrix. This hydration effect slows moisture migration toward the product surface during frying, thereby modifying the rate of vapor escape and crust formation. Consequently, the altered moisture transport dynamics contribute to improved structural stability and reduced oil absorption (62).

Another critical mechanism observed in the reviewed studies is the formation of surface films during frying. As the batter-coated product is exposed to high frying temperatures, alginate-containing formulations can form cohesive surface layers that act as semipermeable barriers (63). These films regulate the exchange of water vapor and oil between the food matrix and the frying medium. By moderating moisture evaporation and oil infiltration, alginate coatings indirectly influence the distribution of flavor compounds and salt within the food structure. This effect plays a particularly important role in modulating salt perception, as localized retention of moisture and flavor molecules can enhance the intensity of taste despite reduced sodium content (64). Collectively, these molecular interactions modify moisture-transport pathways, crust microstructure, and flavor-release kinetics, ultimately contributing to improved sensory perception in reduced-sodium formulations (65). Thus, alginate functions not only as a structural hydrocolloid but also as a functional modulator of mass transfer processes within fried and coated foods.

### 3.6 Batter matrix engineering

The systematic review revealed that alginate-based hydrocolloids play a critical role in engineering the rheological and structural properties of batter systems. Batter formulations serve as complex colloidal dispersions consisting of starches, proteins, hydrocolloids, and water (66). The incorporation of alginate significantly alters these systems by enhancing the viscoelasticity and stability of the batter matrix (65). One of the most consistently reported functional effects across the included studies was a substantial increase in batter viscosity following alginate incorporation. Increased viscosity improves the suspension stability of batter components and enhances the batter’s ability to adhere to food substrates (67). Improved adhesion ensures more uniform coating thickness, which is essential for achieving consistent crust formation during frying. Moreover, higher batter viscosity reduces batter runoff during processing, thereby improving coating efficiency and product yield (68). Another important effect of alginate addition is the improvement of coating adhesion and structural uniformity. The polymeric nature of alginate facilitates interactions with proteins and starches within the batter, promoting the formation of interconnected networks. These networks enhance the cohesiveness of the batter layer and reduce coating defects such as cracks or detachment during frying (69). The reviewed studies also consistently reported significant reductions in oil absorption in alginate-modified batter systems. Reduced oil uptake is primarily attributed to the enhanced structural integrity of the batter film and the formation of more compact crust structures (70, 71). Niklas Lorén et al. (72) investigate lower oil absorption, which is particularly important in the context of sodium-reduction strategies because it can improve nutritional profiles and enhance flavor balance. According to Fang et al. (73), alginate incorporation was associated with improved textural properties, particularly increased crispness and crunchiness of fried products. Instrumental texture analyses showed higher values for hardness and fracturability in alginate-modified systems. These improvements are closely linked to the formation of more stable crust structures and optimized moisture gradients during frying. Rheological measurements further support these observations. Several studies reported increases in both storage modulus (G′) and loss modulus (G″) following alginate addition. The increase in storage modulus indicates enhanced elastic behavior and stronger internal network structures, while the increase in loss modulus reflects improved viscous resistance to deformation (74). Together, these rheological changes confirm that alginate contributes to the formation of robust viscoelastic batter networks, which are essential for maintaining coating stability during frying.

### 3.7 Microstructural barrier effects

Microstructural analyses across several studies, often using scanning electron microscopy (SEM), reveal that alginate-containing coatings produce more compact, less porous crust structures than those of conventional batter systems (75). These structural modifications play a crucial role in controlling heat and mass transfer processes during frying. Alginate-based coatings tend to form continuous polymeric films on the surface of food products. These films reduce the formation of large pores and cracks in the crust layer, resulting in a more uniform, denser microstructure (76–78). The reduction in pore size significantly limits pathways through which oil can penetrate the food matrix during frying.

Additionally, alginate coatings slow moisture evaporation from the interior of the product. This semipermeable barrier formed by alginate films regulates vapor diffusion, maintaining higher internal moisture levels during frying (79). The moisture retention contributes to improved juiciness and prevents excessive dehydration of the food core. The combined effect of reduced pore formation and controlled moisture migration leads to lower oil uptake and improved structural stability of the crust. As oil penetration is minimized, the final product exhibits a lighter texture and improved mouthfeel. At the same time, the stabilized crust microstructure helps preserve the integrity of the coating during post-frying handling and freezing processes, which is particularly relevant for coated frozen food products (7, 80–82).

### 3.8 Quantitative relationships among functional variables

To further explore the relationships among key functional variables reported across the 35 included studies, a Spearman rank correlation analysis was conducted. This non-parametric statistical approach was selected because the extracted data originated from heterogeneous experimental designs and measurement scales across different studies. The correlation analysis should be interpreted as exploratory rather than confirmatory due to substantial heterogeneity in experimental designs, food matrices, hydrocolloid formulations, analytical methods, and reported outcome measures across the included studies. The correlation matrix (Table 4) reveals several statistically meaningful associations between alginate/AOS concentration, batter rheological properties, oil absorption behavior, and sensory attributes of coated fried foods. Prior to analysis, extracted numerical variables such as oil uptake, moisture retention, viscosity, and texture parameters were converted into relative percentage changes compared with corresponding control formulations wherever possible. This standardization approach facilitated cross-study comparison despite differences in measurement scales and experimental conditions. The analysis indicates that alginate/AOS concentration exhibited a strong positive correlation with batter viscosity (*ρ* = 0.63, *p* < 0.001) and storage modulus (G′) (*ρ* = 0.58, *p* < 0.001). These results are consistent with previous research by El Nokab et al. (83), who found that alginate-based hydrocolloids significantly enhance batter viscoelasticity through ionic cross-linking and hydrogel network formation, thereby increasing coating stability and adhesion during frying. Similar rheological behavior of alginate-enriched batter systems has been reported in studies such as Regulation of oil penetration, lipid oxidation, and flavor characteristics in batter-coated fried fish cubes: The functional implications of hydrocolloids by Li et al. (84), which demonstrated that hydrocolloid incorporation significantly increases batter viscosity and storage modulus, improving coating integrity during frying. Conversely, oil absorption showed a strong negative correlation with alginate concentration (*ρ* = −0.62, *p* < 0.001) and batter viscosity (*ρ* = −0.54, *p* = 0.001). These findings support previous observations that hydrocolloid-based coatings act as barrier layers limiting oil penetration during deep-fat frying. The mechanism involves the formation of compact polymer films that reduce crust porosity and slow oil migration into the food matrix. Similar conclusions were reported in sodium alginate-combined ultrasonic modification of starch: Structural characterization, batter properties, and crispiness improvement in fried fish by Li et al. (85), which showed that alginate and other hydrocolloids significantly decrease oil absorption by modifying moisture evaporation and crust structure during frying. In addition, moisture retention demonstrated moderate positive correlations with alginate concentration (*ρ* = 0.55, *p* = 0.001) and crispness score (*ρ* = 0.56, *p* = 0.001). Alginate has a strong water-binding capacity, enabling coated products to retain internal moisture while forming a crispy outer crust. The dual functionality of hydrocolloids in moisture retention and texture development has been widely documented in fried food systems. Varela et al. (86) highlighted the ability of alginate-based coatings to improve moisture management and textural stability in fried products.

**Table 4:** Spearman correlation matrix showing relationships among alginate/AOS concentration, batter rheological properties, oil absorption, moisture retention, crispness, and sensory acceptability across the 35 studies included in this systematic review.

Variable	1	2	3	4	5	6	7
1. Alginate/AOS concentration	1.00						
2. Batter viscosity	0.63 (*p* < 0.001)	1.00					
3. Storage modulus (G′)	0.58 (*p* < 0.001)	0.67 (*p* < 0.001)	1.00				
4. Oil absorption	−0.62 (*p* < 0.001)	−0.54 (*p* = 0.001)	−0.47 (*p* = 0.004)	1.00			
5. Moisture retention	0.55 (*p* = 0.001)	0.48 (*p* = 0.004)	0.51 (*p* = 0.002)	−0.49 (*p* = 0.003)	1.00		
6. Crispness score	0.49 (*p* = 0.003)	0.52 (*p* = 0.002)	0.60 (*p* < 0.001)	−0.53 (*p* = 0.001)	0.56 (*p* = 0.001)	1.00	
7. Sensory acceptability	0.41 (*p* = 0.015)	0.44 (*p* = 0.010)	0.46 (*p* = 0.006)	−0.39 (*p* = 0.021)	0.59 (*p* < 0.001)	0.64 (*p* < 0.001)	1.00

Furthermore, sensory acceptability showed strong positive correlations with crispness (*ρ* = 0.64, *p* < 0.001) and moisture retention (*ρ* = 0.59, *p* < 0.001). These results suggest that improvements in textural properties and moisture balance contribute significantly to consumer perception of product quality. Previous sensory studies have similarly demonstrated that hydrocolloid-enhanced coatings can maintain or even improve crispness and overall acceptability in reduced-sodium fried foods. Such findings are supported by the study by Chaeyoung Jung and Imkyung Oh, titled “Impact of hydrocolloids on oil absorption, oxidative stability, and textural properties of fried fish protein snacks,” which reported that hydrocolloid-enriched coatings improved crispness and consumer preference while reducing oil uptake (87). Collectively, these findings demonstrate that alginate and alginate oligosaccharides act as multifunctional structuring agents in coated food systems. By simultaneously enhancing batter rheology, reducing oil absorption, improving moisture retention, and maintaining desirable sensory properties, alginate-based ingredients provide an effective technological strategy for developing reduced-sodium fried foods with improved nutritional profiles and consumer acceptability.

### 3.9 Sensory compensation strategies

One of the major challenges associated with sodium reduction in processed foods is the loss of flavor intensity and overall consumer acceptability. The results of this review indicate that alginate-based hydrocolloid systems can partially compensate for reduced sodium levels by enhancing several key sensory attributes (72). Firstly, alginate-modified coatings improve crispness and textural contrast, which are critical determinants of consumer satisfaction in fried foods. Crisp crust structures enhance auditory and tactile sensory responses during consumption, thereby positively influencing overall product perception, even when salt levels are reduced (88). Secondly, the moisture-retention capacity of alginate helps maintain the product’s desirable juiciness and mouthfeel. This effect prevents the dryness often associated with reduced-sodium formulations and supports a more balanced sensory profile (89). Another important mechanism involves modulating the dynamics of flavor release. By controlling moisture distribution and microstructural characteristics, alginate coatings influence the release of flavor compounds during mastication (90). The controlled release of flavor molecules can enhance the perceived intensity of saltiness and other taste attributes, thereby compensating for reduced sodium content. Several studies reported that alginate-based coatings successfully reduced oil absorption while maintaining, or even improving, sensory acceptability in fried food products. Sensory panel evaluations frequently indicated comparable ratings for taste, texture, and overall acceptability between reduced-sodium formulations containing alginate and conventional formulations with higher salt levels (91, 92). Furthermore, findings suggest that alginate functions not only as a structural hydrocolloid but also as a sensory optimization tool in reduced-sodium food formulations (93). Through their combined effects on texture, moisture retention, and flavor release, alginate-based systems offer promising strategies for maintaining product quality while supporting public health initiatives to lower dietary sodium intake (94).

## 4 Summary of main findings

This systematic review demonstrated that marine-derived alginate and alginate oligosaccharides (AOS) are effective multifunctional hydrocolloids for sodium-reduced coated frozen foods. Across 35 included studies, alginate-based systems consistently improved batter rheology, coating adhesion, moisture retention, and crust stability while reducing oil absorption during frying. The findings further showed that alginate coatings formed compact semipermeable barriers that controlled moisture migration and limited oil penetration, resulting in denser crust structures and improved textural properties such as crispness and juiciness. Sensory evaluations indicated that alginate-containing reduced-sodium products maintained acceptable flavor, mouthfeel, and overall consumer acceptability, comparable to those of conventional formulations. Quantitative synthesis additionally revealed strong positive relationships between alginate concentration, batter viscosity, moisture retention, and sensory quality, alongside negative correlations with oil uptake. Overall, the evidence suggests that alginate and AOS provide a promising technological strategy for developing healthier, reduced-sodium fried and coated foods without compromising product quality.

### 4.1 Limitations of the systematic review

Several limitations should be considered when interpreting the findings of this systematic review. The included studies exhibited considerable variability in food matrices, hydrocolloid formulations, and sodium-reduction strategies, frying conditions, analytical methods, and reported outcome measures. Owing to this methodological heterogeneity, a quantitative meta-analysis was not feasible, and the findings were synthesized using a qualitative narrative approach. Additionally, although alginate-based systems have been widely investigated, relatively few studies have specifically focused on alginate oligosaccharides (AOS), suggesting that their distinct functional roles in reduced-sodium batter systems remain insufficiently characterized. Methodological quality also varied across studies, with some reports providing limited information regarding experimental randomization, replication, sensory panel design, and intervention standardization. Moreover, the review included only studies published in English-language journals. Consequently, potentially relevant studies published in other languages may have been excluded, potentially introducing language bias and limiting the comprehensiveness of the synthesized evidence. Furthermore, while instrumental analyses such as rheology, texture, and oil absorption were extensively reported, comprehensive consumer-oriented sensory evaluations were less frequently conducted. The molecular mechanisms underlying sodium perception and flavor modulation in alginate-based systems also require further clarification. Finally, the included studies reflected broad international research interest, with major contributions from Asia and Europe alongside studies from other regions; however, broader geographical representation may further strengthen the global applicability of future findings.

### 4.2 Research gaps and future directions

Despite the growing body of literature on alginate-based hydrocolloids in fried and coated food systems, this systematic review identifies several critical knowledge gaps that must be addressed to fully exploit the potential of marine-derived alginate oligosaccharides (AOS) in sodium-reduced food formulations. Although numerous studies have investigated the functionality of conventional sodium alginate in batter and coating systems, relatively few studies have specifically examined the role of alginate oligosaccharides, particularly within the context of sodium reduction in coated frozen foods. This gap is noteworthy because AOS possess unique structural and physicochemical properties, including lower molecular weight, enhanced solubility, and increased bioactivity that may influence their interactions within complex food matrices differently from those of high-molecular-weight alginate polymers. Consequently, further research is required to understand how AOS can be effectively incorporated into batter systems and how their molecular characteristics influence rheology, coating performance, and flavor perception. Another significant gap identified in the reviewed literature is the limited number of consumer-oriented sensory studies evaluating reduced-sodium coated food products. While many experimental investigations have focused on instrumental measurements such as batter viscosity, oil uptake, and texture profile analysis, relatively few studies have conducted comprehensive sensory evaluations involving trained panels or consumer acceptance testing. This limitation restricts the ability to fully assess the practical feasibility of sodium-reduced formulations in real market conditions. Since consumer perception of taste, texture, and overall acceptability ultimately determines product success, future research should incorporate more robust sensory methodologies, including descriptive sensory analysis, consumer preference testing, and cross-cultural perception studies.

Another important research gap concerns the limited mechanistic understanding of sodium ion (Na^+^) interactions and taste-modulation mechanisms in alginate-based systems. Although several studies have suggested that alginate coatings can enhance perceived saltiness despite reduced sodium levels, the underlying molecular mechanisms remain poorly understood. It is hypothesized that alginate matrices may influence ion diffusion, salt distribution, and flavor release kinetics within the food structure. Additionally, interactions between alginate polymers and sodium ions may alter the spatial distribution of salt within the crust and interior matrix, thereby affecting how saltiness is perceived during mastication. However, detailed investigations into these phenomena, particularly at the molecular and microstructural levels, remain limited. Advanced analytical techniques such as nuclear magnetic resonance (NMR), ion-selective analysis, and microstructural imaging could provide deeper insights into the mechanisms governing sodium binding and taste perception. The review also highlights the need for multi-hydrocolloid formulation strategies that combine alginate or AOS with other functional ingredients such as starches, proteins, and dietary fibers. Current research often examines alginate as a single hydrocolloid component; however, food matrices are inherently complex systems where multiple polymers interact synergistically. Combining AOS with complementary biopolymers could enhance batter stability, improve film-forming capacity, and optimize mass transfer processes during frying. For example, starch alginate interactions may enhance viscosity and structural rigidity, while protein-alginate interactions could strengthen gel networks and improve coating adhesion. Similarly, the incorporation of dietary fibers may contribute additional health benefits while further modifying water-binding capacity and texture. Therefore, integrated hydrocolloid systems represent a promising direction for developing advanced reduced-sodium batter formulations with improved technological and sensory properties.

Looking forward, future research should adopt multi-scale and interdisciplinary approaches that integrate molecular chemistry, food microstructure engineering, and sensory science. At the molecular level, investigations should explore the structural properties of AOS, including the degree of polymerization, monomer composition, and ionic interactions with sodium and calcium ions. At the microstructural level, advanced imaging techniques such as scanning electron microscopy (SEM) and confocal microscopy can be used to examine how alginate-based coatings influence crust formation, pore distribution, and moisture migration during frying. Finally, sensory science approaches should evaluate how these structural modifications translate into changes in flavor perception, texture, and consumer acceptability. Overall, addressing these research gaps will contribute to a deeper understanding of how marine-derived alginate oligosaccharides can be strategically utilized in food matrix engineering. By integrating advances in polymer chemistry, food processing, and sensory evaluation, future work can facilitate the development of innovative coating systems that deliver reduced-sodium foods with high sensory quality and nutritional value.

## 5 Conclusion

This systematic review highlights the significant potential of marine-derived alginate and its oligosaccharides (AOS) as multifunctional ingredients for the development of reduced-sodium-coated frozen foods. The collective evidence from the reviewed studies demonstrates that alginate-based hydrocolloids can effectively modify the physicochemical and structural properties of batter systems, thereby addressing many of the technological challenges associated with sodium reduction in fried and coated food products. Alginate functions primarily through structural and mass-transfer mechanisms, including ionic gel formation, water-binding capacity, and the formation of semipermeable surface films during frying. These mechanisms contribute to improvements in batter rheology, coating adhesion, and crust formation, while simultaneously influencing moisture retention and oil migration. As a result, alginate-containing coatings often exhibit reduced oil absorption, enhanced crispness, and improved textural stability, all of which are critical quality attributes in fried foods. Importantly, the reviewed literature indicates that alginate-based systems can also support sensory compensation in reduced-sodium formulations. By enhancing crust structure, maintaining internal moisture, and modulating flavor release dynamics, alginate coatings help preserve desirable sensory characteristics such as crispness, juiciness, and overall palatability. These functional properties enable reduced-sodium products to achieve sensory acceptability comparable to that of conventional formulations, thereby supporting public health efforts to lower dietary sodium intake without compromising consumer satisfaction. Although the broader alginate literature demonstrates strong technological potential, direct evidence regarding AOS-specific applications in sodium-reduced coated frozen foods remains limited and warrants further investigation. Given their unique structural features, such as lower molecular weight, higher solubility, and potential bioactivity, AOS may offer additional functional advantages in food systems. Integrating AOS into batter formulations represents a novel, underexplored strategy for engineering food matrices that deliver improved nutritional profiles while maintaining desirable technological and sensory properties. Future research should therefore focus on interdisciplinary approaches that integrate marine polysaccharide chemistry, food microstructure engineering, and sensory science. Such approaches will be essential for understanding the molecular interactions of AOS within complex batter matrices, optimizing coating performance during frying, and evaluating consumer perception of reduced-sodium products. Additionally, advanced analytical techniques should be employed to investigate the role of AOS in ion transport, flavor release, and microstructural stabilization within fried food systems.

In conclusion, marine-derived alginate and its oligosaccharides represent promising functional ingredients for next-generation sodium-reduction strategies in the food industry. By enabling the design of batter and coating systems that maintain structural integrity, control oil uptake, and preserve sensory quality, these hydrocolloids offer a viable pathway to developing healthier fried and frozen food products that align with global nutritional recommendations for reduced sodium intake.

## Data Availability

The original contributions presented in the study are included in the article/supplementary material, further inquiries can be directed to the corresponding author.
